# Women’s lived experience of well-being in everyday life when living with a stress-related illness

**DOI:** 10.1080/17482631.2020.1754087

**Published:** 2020-04-22

**Authors:** Ulrica Hörberg, Petra Wagman, Anna Birgitta Gunnarsson

**Affiliations:** a Department of Health and Caring Sciences, Faculty of Health and Life Sciences, Linnaeus University, Växjö, Sweden; b Department of Rehabilitation, School of Health and Welfare, Jönköping University, Jönköping, Sweden; c Department of Research and Development, Region Kronoberg, Växjö, Sweden; d Department of Clinical Neuroscience and Rehabilitation, University of Gothenburg, Gothenburg, Sweden

**Keywords:** Exhaustion disorder, health, interviews, mental health, photographs, primary healthcare, reflective lifeworld research, stress-related illness, well-being

## Abstract

**Purpose**: The aim of the study was to describe how women with stress-related illness experience well-being in everyday life.

**Methods**: The study was based on a reflective lifeworld research (RLR) approach and the methodological principles of openness, flexibility and bridling. Twelve women, aged 27–54 years, diagnosed with stress-related illness were included. Data were collected with lifeworld interviews based on photographs taken by the women relating to well-being in everyday life. The data were analysed for meaning.

**Results**: Well-being emerged in situations where women could feel an unconditional beingness. This entails not having demands on oneself and includes some form of freedom from having to perform. The surroundings and supportive environments are important for this unconditional beingness to be present. In order to feel well-being in everyday life, the women need to balance their energy and find helpful tools that can achieve a balance in everyday life.

**Conclusions**: Healthcare staff need to understand the importance of unconditional beingness in supportive environments for patients living with stress-related illness in order to support their health and well-being. It is also important to support patients in finding helpful tools that can aid them to achieve a balance in everyday life.

## Introduction

This study focuses on well-being among Swedish women living with a stress-related illness, such as exhaustion disorder or a reaction to severe stress. Health, well-being and positive experiences of physical, mental and social status are of importance for a healthy life (World Health Organization [WHO], 2019), even though it may be difficult to have a sufficiently healthy everyday life when suffering from illnesses. Using “positive” qualities in life, such as strengths, attitudes and emotions that can enable the individual to grow and develop, is also of importance for enhancing well-being and not just the reduction of symptoms of illness (Seligman, 2017). Developing strengths and using them in everyday life can promote an active participation in everyday life, not only in physical terms but also mentally, socially and culturally (Iwasaki et al., 2006). Dahlberg et al. (2009) describe an existential and lifeworld-oriented view of well-being, including existential dimensions of freedom and vulnerability. In addition, the cornerstones of health and well-being are vitality, movement and peace, which could be further described in terms of life rhythm, i.e., finding harmony and balance as well as both stillness and movement in one’s life (Dahlberg et al., 2009). Todres and Galvin (2010) have developed an existential theory of well-being termed “dwelling mobility”, based on continental philosophy, which they maintain has the greatest potential for existential beingness. Dwelling mobility can be described as an “adventure” with existential possibilities and also as “being at home with”, including meanings of rootedness and flow, peace and possibility.

The most common reasons for sick leave among women in Sweden are often musculoskeletal pain or stress-related illness (Osika Friberg et al., 2016). Mental health disorders, such as depression and anxiety disorders, and stress-related illnesses are the most common causes of long-term sick leave among both men and women. Furthermore, stress-related illnesses are, compared to other disorders, those that are increasing the most among women in Sweden and many other Western countries (Försäkringskassan, 2018, 2014; SBU, 2015). Stress-related illnesses, with physical as well as psychological symptoms, e.g., muscle pressure, depressed mood, anxiety, insomnia, and tiredness (Socialstyrelsen, 2003), often affect the individual’s ability to cope with different activities in everyday life and to manage social relationships (Håkansson & Ahlborg, 2017). This may also cause an individual to be put on sick leave and who then can have a long struggle to return to work (Försäkringskassan, 2017). This is also related to occupational balance, i.e., people’s perception of the amount of and variation between their activities in everyday life (Wagman et al., 2012). Occupational balance has been positively related to health and well-being (Wagman & Håkansson, 2014), negatively related to stress (Yu et al., 2018) and an imbalance may contribute to stress-related illnesses (Håkansson & Ahlborg, 2018). Perceptions of occupational imbalance have been found to be associated with perceived stress in both women and men (Håkansson & Ahlborg, 2017). Similarly, life balance has been shown to be related to perceived stress (Matuska et al., 2013).

Patients suffering from stress-related illnesses in Sweden can be offered a number of interventions (Bergström et al., 2015; Försäkringskassan, 2014; Holmgren et al., 2016; Pálsdóttir et al., 2014). There is, however, a knowledge gap concerning which of these provides the most effective treatment (SBU, 2015), and there is a need for research that focuses not only on symptoms but also on evaluating interventions, which are preventive and promote the individual’s return to work (FORTE, 2015). Interventions developed for patients with mental health disorders, provided by the healthcare services as well as the occupational health service, have shown moderate effects (Bergström et al., 2015). An interview study with women suffering from stress and burnout highlighted aspects that promote resilience and rehabilitation. The results revealed a progression, from needing social support from professionals and families, being aware of the women’s needs and limitations, gradually regaining joy and interest, to finally enhancing well-being, and being able to work and be in a social context (Salminen et al., 2015). The aforementioned studies indicate a need for developing and evaluating additional methods that can promote well-being, health and a return to work.

Activities, which are performed solely because they are desired, e.g., doing something creative or physical that is considered enjoyable by the doer, have been shown to be important for health and well-being in women in working age, recovering from stress-related illnesses (Håkansson et al., 2006). Furthermore, enjoyable activities have proved to be relevant for rehabilitation with women in working age, in connection with stress-related illnesses (Eriksson et al., 2011; Håkansson et al., 2006; Johansson et al., 2012). For example, the enjoyment of gardening in a gardening rehabilitation programme was stated as being important and the participating women emphasized the need to re-implement enjoyable activities in their everyday life (Eriksson et al., 2011). Meaningful and pleasurable leisure activities have also been described as having great value and as being health-promotive to people with mental illness (Iwasaki et al., 2010). Such activities have also been found to be of importance in an overview focusing on quality of life in mental illness (Connell et al., 2012). These activities can be experienced as energy-intensive as well as restful (Johansson et al., 2012) and perceived as non-demanding and thus generating a feeling of freedom (Eriksson et al., 2011; Håkansson et al., 2006). We have thus found research reporting the experiences of stress-related illness in relation to occupational balance and the consequences of occupational imbalance and to social relationships in everyday life. We have also found that rehabilitation can facilitate a progression from needing support from others to having their well-being in their own hands. We have, however, not found research that specifically focuses on well-being per se when living with stress-related illness. Such knowledge could be of importance in the planning of interventions that contribute to recovery from stress-related illnesses. Further research is thus required in order to gain a deeper understanding of the meaning of living with stress-related illness, including exploring whether it is possible to experience well-being in spite of a stress-related illness, and what is associated with well-being.

## Aim

The aim of the study was to describe how women with stress-related illness experience well-being in everyday life.

## Method

### Design

This study is based on a reflective lifeworld research (RLR) approach (Dahlberg & Dahlberg, 2019a; Dahlberg et al., 2008). RLR is founded on phenomenology, the continental philosophy of Husserl’s lifeworld theory (Husserl, 1936/1970) and the theory of intentionality (Husserl, 1929/1977) as well as Merleau-Ponty’s theory of the lived body (Merleau-Ponty, 1945/2011) and the ontology of the “flesh of the world” (Merleau-Ponty, 1964/1968). The goal is to understand and describe the meanings of the phenomenon: well-being in everyday life when living with a stress-related illness.

The methodological principles of RLR are openness, flexibility and bridling (Dahlberg & Dahlberg, 2003; Dahlberg et al., 2008). The researcher needs to adopt a bridled (reflective) attitude throughout the research process in order not to understand the meaning of the phenomenon too quickly and un-reflected. It means to be open and flexible to the phenomenon and at the same time slowing down the process of understanding.

### Participants and study setting

Twelve women, aged 27–54 years with a mean age of 39 years, and diagnosed with stress-related illness according to ICD−10, F43.8—other reactions to severe stress and F43.9—reaction to severe stress, unspecified (WHO, 2004), were included in the study. They were recruited with the help of staff from three primary healthcare centres in two counties in the south of Sweden (see Table I for characteristics of the informants). Firstly, an individual information meeting, with the first or last author, took place at the healthcare centre or at another place chosen by the presumptive informant. Verbal and written information about the study including that the interview would be based on photographs was provided at this meeting.

**Table I.** Characteristics for the participants (n = 12)

**Gender**	
Women/Men	12/0
**Age**	
Mean(SD):	39.5(10.7)
Min-Max	27, 54
**Living status**	
Single	5
With someone	7
**Have children ≤ 18 years**	5
**Educational level**	
University	7
High school degree	5
**Main support**	
Employed/Student	10/1
Unemployed	1
**Sick-leave** yes/no:	10/2
**Primary diagnosis**	
Other reactions to severe stress (F43.8)	11
Reaction to severe stress, unspecified (F43.9)	1

### Data collection

Data were collected with a combination of lifeworld interviews (Dahlberg et al., 2008) and photographs taken by the women. The photovoice methodology was the inspiration for the use of photographs (Hansen‐Ketchum & Myrick, 2008; Wang & Burris, 1997). The informants took photographs with their own mobile phones or digital camera in order to stimulate the informants’ reflections about well-being in everyday life.

Prior to the interview at the first information meeting, each informant was asked to take approximately three photographs of situations and moments in their everyday life when they experienced well-being. They were also informed to email the photographs before the interview. All the interviews, which were audio-recorded, were conducted by the first or last author at a place chosen by the informants and lasted between 60 and 90 minutes. Prior to the interviews, the interviewer had printed the photographs on A4-format and these formed the starting point for the interview. The focus of the women’s photographs were, for example, nature, their pets, things, the environment and situations indoors or outdoors, alone or together with others. Each informant was asked to choose which photograph she wanted to start talking about and the interview started with the following opening questions “Please, tell me about the photograph, what it is about?”, “Can you please describe how the photograph relates to well-being in your everyday life?” and “What kind of emotions does the photograph evoke?” To gain a deeper understanding of the lived experiences of the phenomenon, follow-up questions were asked, such as “Can you give me an example of well-being in your everyday life?”, “Can you tell me more about this?” The same questions were used for all the photographs.

### Data analysis

The data were analysed for meaning in accordance with the RLR principles of openness, flexibility and bridling (Dahlberg & Dahlberg, 2019a, 2019b; Dahlberg et al., 2008). The analysis was characterized by a movement between the whole—the parts—to a new whole in order to describe the phenomenon’s essential structure of meanings. Firstly, the interviews were transcribed verbatim into text. The analysis started with a search for meanings in data of the phenomenon “well-being in everyday life when living with a stress-related illness”. Furthermore, the meanings were related to each other based on similarities and differences and grouped into clusters. The analysis continued by searching for patterns of meanings in-between the clusters. In order to understand how the clusters were related to each other and, in line with the principles of RLR, questions were asked to the data such as: How does well-being in everyday life occur when living with stress-related illness? What does well-being mean in everyday life when living with stress-related illness? The phenomenon’s essential structure of meanings gradually emerges during the analysis process as well as the more contextual nuances of meanings called constituents. Openness and flexibility towards the phenomenon were also sought during the analysis together with a reflective bridled attitude, with the purpose of not defining and denominating too quickly (Dahlberg et al., 2008).

## Ethical considerations

This study was approved by the Regional Ethical Review Board of Linköping University, Sweden (Reg. no. 2017/284-31). The informants were informed both verbally and in writing about the aim of the study, the voluntary participation and the confidential handling of their photographs, what they said and any background information they provided. All interview transcripts were anonymized immediately after data collection. All of them gave their verbal and written informed consent to participate in the study.

## Results

The result is first presented by the essential structure of meanings, followed by its constituents.

The core element for attaining well-being in everyday life when living with a stress-related illness is a space for unconditional being. This is intertwined with not having demands and embraces some form of freedom from having to perform individually or in an interpersonal context. The surroundings and the atmosphere therein are important for the unconditional to present itself. In order for well-being to emerge, space is needed for privacy and tranquillity where there are no demands and batteries can be recharged.

Energy is replenished by meaningful activities and companionship in everyday life. However, existence is complex when the activities that provide well-being simultaneously also consume energy. The possible well-being competes with the obligations and demands that cannot always be opted out from but have to be dealt with. This entails being forced to struggle for well-being where the will exists, and energy is sought at the same time as fatigue impedes. There is a limit for what can be managed, and this limit is difficult to identify and easy to overrun, leading to health consequences. This demands a need to make the “right” choices and carefully balance whether well-being in the moment is worth its price, being drained of energy. Moments that give enjoyment can at the same time be precarious and need a long recovery. Time for recovery thus needs to be planned and accepted.

Well-being presents itself when life moves in a direction where “light can be seen”. The light can be faint, but still visible, and it is the small changes that make a difference, such as being able to shift focus from oneself to others or other things or other situations and at the same time being able to balance existence and recovery. In order to do this, it is necessary to be able to have control over one’s time and life situation and to find the tools that can balance the existence. Possibilities for resting in activities or resting in beingness are recovering. There is no disparity between rest and activity, and when resting in activity presents itself then well-being also presents itself.

The unconditional being in nature has a curative effect. Nature provides freedom far from demands and obligations, where seeing it and being in it generate well-being. External support, in the form of understanding, attention and sufficient pushing to find balance and manage everyday life, is important in the movement towards well-being and recovery.

The following constituents further illuminate the meaning of the phenomenon: *The unconditional beingness, The important energy that is so difficult to balance, Tools that help to achieve a balance in everyday life* and *The supportive environment*.

### The unconditional beingness

Space for unconditional being is required in order to feel well-being in everyday life when living with a stress-related illness. It is important just to be able to relax and to leave things that otherwise might be disturbing. The unconditional beingness appears when opportunities for privacy exist and there are possibilities to be by oneself in peace and quiet and be able to focus on oneself and one’s own well-being. It may be necessary to shield oneself from one’s surroundings in order to achieve relaxation.

The bathroom is … a protected zone or a safety zone. // When I’m having a shower at night … then everything disappears … the feelings of stress and a sense of time and … It’s so good … the warmth, having a shower, lathering myself and putting on lotion afterwards … and then … then … it’s almost meditative … that I can just be … (5)

The unconditional beingness appears in situations and/or environments that do not require having to perform or achieve something. When living with stress-related illness, it is important to be able to control one’s own time and situation and to be able to do things that make one feel well at one’s own pace. It is also important to be able to change focus to something other than oneself. Environments that support unconditional beingness can be places in the home where there is an atmosphere that provides well-being. They can also be places outdoors such as a garden, a library or a special place by a lake. These environments contribute to feelings of being free from demands, musts and having to perform.

You don’t need to perform // … I love going to the library, It’s the best, you walk in and then … sigh … wonderful, calm, and just walk around and browse among the books and see what can be borrowed … it’s like stepping into a new world, then it’s just as though all the pressure disappears … (9)

Opportunities for unconditional beingness are provided when one is together with people with whom one feels safe and there is an opportunity to be oneself without feeling demands to perform and of having to be in a particular way. *Meeting one’s family and not feeling pressured to do anything, it’s like … all the musts are put aside // we can laugh and … sometimes not talk at all and just be … it’s very enjoyable … (12)*

### The important energy that is so difficult to balance

Well-being in everyday life when living with stress-related illness entails a struggle to feel well, which is shown in the contradiction between the desires on the one hand and the lack of energy on the other.

Coping with this everyday existence is difficult due to the situation around the person not being adapted to her levels of strength and energy. It thus becomes necessary to find balance in everyday life and to use the energy for things that provide strength and well-being, which thus means trying to make the right choices in everyday life. At the same time, it entails having to face the consequences of being drained of energy in order to feel well-being and enjoyment in the moment. Furthermore, it can be difficult to know in advance what the consequences of the choice will be and whether it is worth the price.

The problem is often … that it can give me pleasure, but takes a lot of energy … // on the one hand I feel that meeting up with friends is great and a lot of fun … but then I know that I’ll be exhausted the day after … (5).

Achieving balance in everyday life requires searching for balance between being in social situations that give enjoyment and situations that generate calm and tranquillity in order to be able to recover. *“I can be very social and enjoy being with people, but later on I need to be by myself to be able to charge my batteries” (7)*. It is, however, difficult to achieve balance between activity and rest and it is easy to get into situations where it becomes too much. It can also vary from time to time as to how many impressions one can take in and how much effort one can manage. *“Sometimes it’s really given me a lot of energy … but sometimes it’s been too much, and then you’ve become exhausted instead … ” (12)*. Trying to see possibilities in one’s life situation can thus mean a struggle for well-being. It sometimes means forcing oneself to do things that one knows provides a temporary sense of well-being, while at the same time knowing there is an imminent risk for setbacks.

One way of achieving well-being in everyday life is by trying to actively and consciously balance one’s energy by calming down and giving space to oneself and one’s needs. For example, *”by allowing … yourself time, like it’s your choice to rest. To actively choose … to rest …” (3)*. It can also entail being able to do things at one’s own pace without being interrupted by, for example, tiring everyday chores.

### Tools that help to achieve a balance in everyday life

It is important to find supportive tools to be able to feel well-being in everyday life when living with a stress-related illness. The tools can be other people who give support, they can also be an inner strength and previous experiences of feeling well that come back and generate feelings of confidence for the future.

It means seeing and feeling that one’s life moves in the direction where light can be seen and where the illness begins to recede and spaces may appear for feelings/emotions that have previously been constrained by stress-related illness.

But now I’m finally there … now I feel a sense of well-being from time to time … I’m not there yet … but it feels like I’ve come out on the other side … in many areas that weren’t possible before … // … it’s a little bit like a feeling of euphoria (3).

The tools are by no means self-evident, but they entail testing and searching to find new ways to deal with everyday life. It requires being thoughtful and having the energy to feel the way to find something that can generate well-being. *”For me, it’s a challenge just to be satisfied with what I actually managed to do today” (3)*. This can entail structuring the everyday life by, for example, writing notes about what to do during the day to make sure that each day contains certain recurring elements. *“Here are the things I want to do every day, exercise, resting, getting up at a specific time …” (3)*. This is described as a help to balance one’s existence in the direction of recovery, and the tools can be finding one’s own moments. This may be seen more profoundly as zones for recovery, where a conscious search for situations that one knows can provide well-being. It can be a major difference if it is done individually or together with others.

I enjoy a coffee break the most if I’m alone. // There’s no one that I imagine could change that, during my coffee break I don’t have the radio on, it’s just quiet. I can have my thoughts for myself and don’t get interrupted. That’s the difficult part for me, being interrupted. Especially considering my exhaustion. If I’m interrupted with, for example, a question I lose my concentration. So, I want this moment for myself … (1).

Well-being can emerge in creative activities where the creativity is given space. It can, for example, be activities such as knitting, painting or making flower and plant arrangements. The women describe this situation as being “one” with the activity and that the “product” (e.g., a painting) gives something back, i.e., the well-being of being able to enjoy the painting. The creative situations, where well-being emerges, are described as the focus when it is directed towards something other than oneself.

… for me, painting is an opportunity to detach myself from my surroundings. It’s like therapy. But I feel like I don’t do it often enough. I want to do it but at the same time there’s some resistance. But when I actually start painting all the pressure goes away, it’s the path leading up to that moment that is filled with a sense of performance. But when I’m there … I just start to paint not knowing … what I’m about to paint (1).

It is about being able to rest in activities by allowing thoughts and emotions to disappear and just be directed in the activity such as yoga, meditation or reading. The common denominator for the activities that give rest is that they take place in a calm atmosphere and/or in privacy. It means being able to relax, to be present and to let one’s thoughts wander.

But then I go up to my bedroom, put on some relaxing music, light some candles and do yoga … I often think that it will take about thirty minutes, but almost every time it goes on for a full hour … and when I’m done … I feel like a new person // I feel much calmer and I feel prepared to face … what every everyday life throws at me (5).

### The supportive environment

The supportive environment is described as being important for finding well-being in everyday life when living with stress-related illness. The supportive environment consists of nature, animals and close trusting relationships that are far from any demands and obligations. Nature is important for attaining well-being, but a secluded place for beingness in the garden is also described as stress-relieving and freedom-giving in contrast to busy and disturbing environments that are avoided in order to find calm.

There is an attraction to nature but it is not always a conscious decision to, for example, go out into the forest, but when they are there the health-promoting properties and positive impact on health are discovered. *”Being by the water has a calming effect on me …” (12)*. Nature provides strength both in solitude and in companionship if there is space for tranquillity. Special places and environments are described as generating feelings of freedom and possibilities for strengthening one’s well-being. Nature is described as curative when it provides beingness in tranquillity and freedom.

I discovered a whole new dimension in nature, I went to my partner’s place where I could just sit … it was calm and quiet and the nature had a healing effect … it was so good … to just be in that moment. But … I still feel like it’s really magical to get out in nature, I don’t know, I can’t really explain, but it gave me an opportunity to heal … (6).

Animals, such as dogs or cats, can by their affection and unconditional love contribute to well-being, especially in situations where there is no strength left for being together with people. The companionship with the animal generates a sense of freedom far from demands even if the pet needs care.

The dog loves me, he’s always curled up next to me and wants to be close … And it’s so cozy. And … you can feel the warmth from his head and his paws. And just at that moment I feel … oh … oh … so wonderful it is right now … (3).

Important people, such as family, grandchildren, friends and workmates, contribute to well-being in everyday life. Being together with others such as these provides possibilities for being listened to and socializing unconditionally. It is essential to be understood and accepted despite them lacking strength. It is also important to be supported in order to manage tasks that can give well-being as well as to feel that there are close ones, who are there, supporting and listening, despite how difficult everyday life is.

She means a lot to me … it’s the kind of girl that I feel I will always be together with, or at least I hope so … a girl that I feel safe with, who listens and gives advice and … I hope that I’m that kind of friend to her as well … yeah … it feels good and secure (6).

## Discussion

This study focused on women’s lived experience of well-being in everyday life when living with a stress-related illness. The study focus was on what generates well-being in life instead of focusing on the obstacles in life. Most of the women expressed during the interviews that they had not previously thought about the phenomenon in focus for this study and had not either been asked any questions about it in connection with appointments at the healthcare services. The women had instead been asked questions about what they were unable to do because of their stress-related illness and had also received advice from the healthcare staff about what to avoid in everyday life.

The results in this study show, from the perspective of women interviewed in this study, how it is possible to feel well-being in everyday life when living with stress-related illness. Feelings of well-being emerged in situations and moments of unconditional beingness and in supportive environments. To feel well-being requires tools for achieving balance and energy in everyday life activities. Being interviewed was experienced as an opportunity to reflect on and to be aware of what well-being meant. To become aware of the meaning of well-being might be seen as a step towards using “good” qualities in life, i.e., strengths, that enabled them to enhance well-being, as proposed by Seligman (2017).

Another tool to enhance well-being was described in the informants’ description on how they could be one with an activity, e.g., painting. This is similar to the experience of flow, i.e., to be completed absorbed in an activity, previously emphasized as essential for enhancing well-being (Csikszentmihalyi, 1990; Engeser & Schiepe-Tiska, 2012). In the present study, experiences of well-being also emerged in situations where the women could feel unconditional beingness. This is similar to another perspective of the flow theory (Jonsson & Persson, 2006) that not only highlights the importance of experiences of flow but also of calming dimensions. Both these perspectives are important for enhancing well-being and balance in everyday life (Jonsson & Persson, 2006), and in this study, the calming, or unconditional beingness seemed to be crucial for health and well-being.

In order to feel well-being in everyday life, the women need to balance their energy and found helpful tools that can achieve balance in everyday life. These results concur with those of Arman et al. (2011), who addressed existential dimensions of burnout with a focus on patterns of health, suffering and expressions of life. The results also correspond with those from studies focusing on life balance in women recovering from stress-related illness (Håkansson et al., 2006) and in “healthy” people, i.e.,, without a recent long-term sick leave (Wagman et al., 2011). Arman et al. (2011) show that the participants in their study search for a “way out” where they could just be and rest in a condition of pure being. The pure being emerges in self-chosen activities like reading a book or being in nature. The results also show that professionals and relatives were important in the search for balance in everyday life, both in terms of pushing forward and setting limits. The finding by Arman et al. (2011) concerning the meaning of “pure being” is similar to the finding in the present study about the meaning of unconditional beingness for well-being in everyday life.

Galvin and Todres (2011, 2013) propose an existential theory of well-being as “dwelling mobility” and mean that well-being is possible irrespective of living with health and/or illness and that well-being can be seen as a resource for both health and illness. Our study shows the importance of finding balance in everyday life, which includes resting both in activities and in beingness in order to balance one’s energy. This finding can be further understood in the light of Galvin and Todres’ description of embodied mobility and dwelling as vitality and comfort (Galvin & Todres, 2011, 2013). Similarly, Dahlberg et al. (2009) and Dahlberg (2011) provide an existential and lifeworld-oriented view of well-being that includes vitality, movement and peace. Even if the women in the current study suffer from stress-related illness, the result shows possibilities for well-being and rest in activities. This can theoretically be understood as peace and movement being intertwined and being of each other. Dahlberg et al. (2009) also describe the importance of finding one’s own life-rhythm between stillness and movement as well as the importance of having minor or major “life projects” that support health and well-being. For example, a life project supporting well-being for the women in the present study could be to find sufficient space for important activities in their everyday lives. This is similar to striving towards occupational balance, i.e., a good mix of everyday activities (Wagman et al., 2012). A major life project for the women in our study was to find the elusive balance in life that contributes to a sustainable life situation and this life balance included, as Håkansson and Ahlborg found (2017), a balance between doing things for your own sake and doing things for others.

The results showed that various tools can constitute support for enhancing well-being, e.g., activities such as creative activities, physical exercises, reading books, and not the least coffee breaks. Spending time in these kinds of activities could be linked to experiences of flow, and of exacting/challenging and calming dimensions (Jonsson & Persson, 2006), which enhanced their experience of well-being. Creative activities, such as doing handicrafts were also found to reduce stress and promote well-being in the present study, which was in line with findings from a study by Pöllänen (2015), where handcrafts provided recreation and supported women to cope with their feelings. Furthermore, reading books was a possibility for resting in an activity and being with oneself, which was experienced as well-being in this study. A similar result was also demonstrated in a study in which women who were on sick leave due to physical or mental disorders were reading and experienced a private zone in which they could enter a fictive world and relate to fictive others (Mårtensson & Andersson, 2015).

Our results show how a supportive environment such as being in nature, for example, in the forest, the garden or by the lake generates well-being both by oneself and together with relatives or pets. The women describe such environments as supportive for well-being and recovering. The benefits from being in a forest environment can be related to the findings in a literature review evaluating the physical and psychological health and well-being benefits of forest therapy or exposure to nature (Oh et al., 2017) as well as the findings on effects of forest bathing (Furuyashiki et al., 2019). These findings support the premise that being in a forest environment may provide benefits when living with stress-related illness.

Sahlin et al. (2015) explored the effects of nature-based rehabilitation for people suffering from stress-related illness. Their results showed increased well-being scores and a reduction in healthcare consumption as well as reduced scores on burnout, depression and anxiety. These results support those in our study where being in a supportive environment, such as a garden or in nature supported women’s experiences of well-being.

Furthermore, two longitudinal studies have focused on therapeutic garden rehabilitation for people with stress-related illness (Eriksson et al., 2011; Sidenius et al., 2017). Eriksson et al. (2011) describe how women on sick leave with stress-related illness experience the rehabilitation in a therapeutic garden. The findings highlight how the women felt more relaxed in the garden and it provided feelings of enjoyment that also inspired the women to add enjoyable activities in everyday life. The women described feelings of freedom and possibilities for being unconditionally creative, for example, making flower arrangements. The described enjoyment can be understood as an expression of well-being as described in our study. Sidenius et al. (2017) have described the lived experience of nature-based therapy in a therapy garden as feelings of familiarity with the garden and a sense of belongingness. The garden was described as a supportive environment and a suitable shelter that contributed to senses of safety and freedom.

Finally, supportive environments also included close trusting relationships and the importance of these has been previously emphasized in women recovering from stress (Håkansson et al., 2006) and in healthy working people (Wagman et al., 2011).

Altogether, our results support those mentioned above, showing that being in nature has a curative effect and provides freedom that is far from demands. They also highlight the importance of a healthy life rhythm in order to find harmony and balance in life, which is in line with the description of an existential view of well-being by Dahlberg et al. (2009).

## Methodological considerations

The methodological principles in RLR—openness, flexibility and a bridling attitude (Dahlberg et al., 2008) were applied throughout the research process in order to achieve objectivity and not taking for granted what is unknown. The research group has with a reflective attitude continuously discussed the emerging results throughout the analysis process. The fact that the results are on a meaning level and describe the phenomenon on an essential level could strengthen the validity of the study.

RLR is an approach without rigid methodological steps (Dahlberg et al., 2008) thus allowing methodological creativity. The use of photographs as a foundation for lifeworld interviews in the data collection is not common. Using photographs can possibly be questioned related to the search for the informants’ more unreflective experience of the phenomenon in focus for the study. Nevertheless, some phenomena are difficult to capture and thus require an openness regarding how to approach the phenomenon under study. Well-being is an abstract phenomenon to grasp. However, the possibility for the women to take photographs of moments and situations of well-being in everyday life was described by the women as an opportunity to identify well-being in their everyday life. The interviews started in the photographs and the focus for the interviews was on the women’s lived experiences of the phenomenon, which were deepened during the interview with an openness and flexibility towards the phenomenon. Several women described that the interview gave a deeper understanding of what generates well-being in everyday life. Moreover, other studies based on a RLR approach have used photographs (e.g., Olausson et al., 2013) or photo cards (e.g., Larsson et al., 2013) as support for data collection in order to deepen the understanding of the phenomenon.

The use of a reflective lifeworld research approach can be seen as a strength in the study due to the abstract level of the essential structure of meanings in the results. According to Van Wijngaarden et al. (2017), the essential level of the results generates possibilities for transferability of the results to similar areas.

It was initially planned that both genders should be included. However, the gatekeepers only managed to recruit women, which may reflect the fact that more women than men suffer from stress-related illness (Försäkringskassan, 2018; SBU, 2015). It is possibly that other or more variations of meanings of the phenomenon under study could emerge in the results if men had also been included in the study. However, on a general level, the essential structure of meanings in the results can be transferred with caution to men but further research is warranted including men as well as women.

## Conclusion

In conclusion, the results show that it is possible to experience well-being in everyday life despite living with stress-related illness and that moments of unconditional beingness in supportive environments are important. Healthcare staff thus need to understand the importance of unconditional beingness (e.g., the importance of feeling an opportunity to just be) in supportive environments for patients living with stress-related illness in order to support their health and well-being as well as their recovery process. It is also important to support patients to find helpful tools that can contribute to achieving a balance in everyday life. In order to further explore how and whether well-being in everyday life can be increased in people living with stress-related illness, the next step is an intervention using photographs as a supplement to treatment as usual in the primary healthcare services for men and women with stress-related illnesses.
